# Anterior cervical discectomy and fusion, open-door laminoplasty, or laminectomy with fusion: Which is the better treatment for four-level cervical spondylotic myelopathy?

**DOI:** 10.3389/fsurg.2022.1065103

**Published:** 2023-01-09

**Authors:** Huajian Zhong, Chen Xu, Ruizhe Wang, Xiaodong Wu, Huiqiao Wu, Baifeng Sun, Xinwei Wang, Huajiang Chen, Xiaolong Shen, Wen Yuan

**Affiliations:** Department of Orthopaedic Surgery, Changzheng Hospital, Naval Medical University, Shanghai, China

**Keywords:** anterior cervical discectomy and fusion, open-door laminoplasty, laminectomy with fusion, cervical spondylotic myelopathy, cervical surgery

## Abstract

Four-level cervical spondylotic myelopathy (CSM) is a common disease affecting a large number of people, with the optimal surgical strategy remaining controversial. This study compared the clinical outcomes, radiological parameters, and postoperative complications of primarily performed surgical procedures such as anterior cervical discectomy and fusion (ACDF), open-door laminoplasty (LAMP), and laminectomy with fusion (LF) in treating four-level CSM. A total of 116 patients who received ACDF (38 cases), LAMP (45 cases), and LF (33 cases) were followed up for a minimum of 24 months were enrolled in this study and retrospectively analyzed. Clinical outcomes were evaluated using the Japanese Orthopedic Association (JOA) scoring system, the Neck Disability Index (NDI), and the Visual Analogue Scale (VAS). Changes in the curvature of the cervical spine were determined using the cervical curvature index (CCI) and the C2–C7 Cobb angle. Cervical mobility was evaluated using the C2–C7 range of motion (ROM) and active cervical ROM (aROM). Complications were recorded and compared among the three groups. All patients achieved significant improvement in JOA, NDI, and VAS scores at the final follow-up (*P* < 0.05), whereas no remarkable difference was found among the groups (*P* > 0.05). In addition, both C2–7 ROM and aROM were significantly reduced in the three groups and LAMP showed the least reduction relatively. As for complications, LAMP showed the lowest overall incidence of postoperative complications, and patients in the ACDF group were more susceptible to dysphagia, pseudoarthrosis than LAMP and LF. Considering improvements in clinical symptoms and neurological function, no remarkable difference was found among the groups. Nevertheless, LAMP had advantages over the other two surgical procedures in terms of preserving cervical mobility and reducing postoperative complications.

## 1. Introduction

Cervical spondylotic myelopathy (CSM) is a progressive, degenerative disease that ranks as the leading cause of spinal cord dysfunction in the adult population (1). The pathogenesis of CSM is characterized by a degeneration of various elements of the cervical spine, such as the cervical vertebral body, intervertebral disc, surrounding ligaments, and accessory structures, which leads to spinal cord or nerve root compression and corresponding neurological symptoms (2). Although conservative treatment shows promising effects for patients with mild symptoms, surgical intervention remains a better option for those with moderate to severe neurological symptoms.

Surgical management of CSM could be achieved through anterior, posterior, or a combined procedure if necessary. The anterior surgical procedure mainly includes anterior cervical discectomy and fusion (ACDF) (3), anterior cervical corpectomy and fusion (ACCF) (4), and cervical disk arthroplasty (CDA) (5); In contrast, laminectomy with or without instrumented fusion and open-door or French-door laminoplasty represent popular posterior surgical procedures (6–8). Due to concerns involved in multilevel surgical management, such as postoperative cervical deformity and segmental instability, ACDF and laminectomy with fusion (LF) are the commonly performed fusion surgeries for multilevel CSM, which are complemented by non-fusion open-door laminoplasty (LAMP), because certain reports indicate that LAMP results in a higher magnitude of function recovery and symptomatic alleviation than French-door laminoplasty (FDL) (9). Up to now, ACDF, LAMP, and LF have been the most commonly performed spinal surgical procedures for multilevel CSM because of their relatively low complication rates and fair neurological outcomes, whereas which among these three is the optimal procedure remains controversial. Although studies comparing the clinical outcomes of these surgical procedures in three-level CSM have been undertaken, there are few reports on four-level CSM. Thus, in the present study, we compare the clinical outcomes of ACDF, LAMP, and LF in treating four-level CSM.

## 2. Materials and methods

### 2.1. Patients

All study procedures were approved by the institute chancellor's Human Research Committee in accordance with the institute's protocol. Ethical approval of this retrospective study was given by the Naval Medical University ethics committee review board. The design and reporting were performed in accordance with the Strengthening the Reporting of Observational studies in Epidemiology (STROBE) statement. This research was conducted in accordance with the Declaration of Helsinki. This study retrospectively reviewed patients who were diagnosed with CSM between February 2008 and January 2014 in our institute, and all patients presented with symptoms of cervical myelopathy with/without radiculopathy. The inclusion criteria were as follows: (1) magnetic resonance imaging and x-ray radiography showing signs of intervertebral disc degeneration or herniation of four consecutive levels; (2) patients diagnosed and suffering from CSM symptoms requiring surgical treatment; and (3) patients treated with either ACDF, LAMP, or LF. The exclusion criteria were as follows: (1) ossification of the posterior longitudinal ligament (OPLL), (2) severe kyphosis, (3) motor neuron disease (MND), (4) previous cervical surgery, (5) history of rheumatoid arthritis, (6) cerebral palsy, (7) thoracic spondylotic myelopathy, (8) lumbar spinal canal stenosis, (9) congenital deformities, and (10) tumors, and trauma. After selection, we included 158 patients and grouped them as ACDF, LAMP, and LF according to the surgical procedure that they underwent. Of the 158 patients, 116 who were followed up for more than 24 months were enrolled (follow-up rate, 73.4%), the remaining 42 patients lost contact during follow-up, and the final sample comprised 60 male and 56 female patients (with a mean age of 56 years; and range of 47–49 years) who were followed up for an average period of 39.4 months (range 24–72 months).

### 2.2. Surgical technique

**Figure 1:**
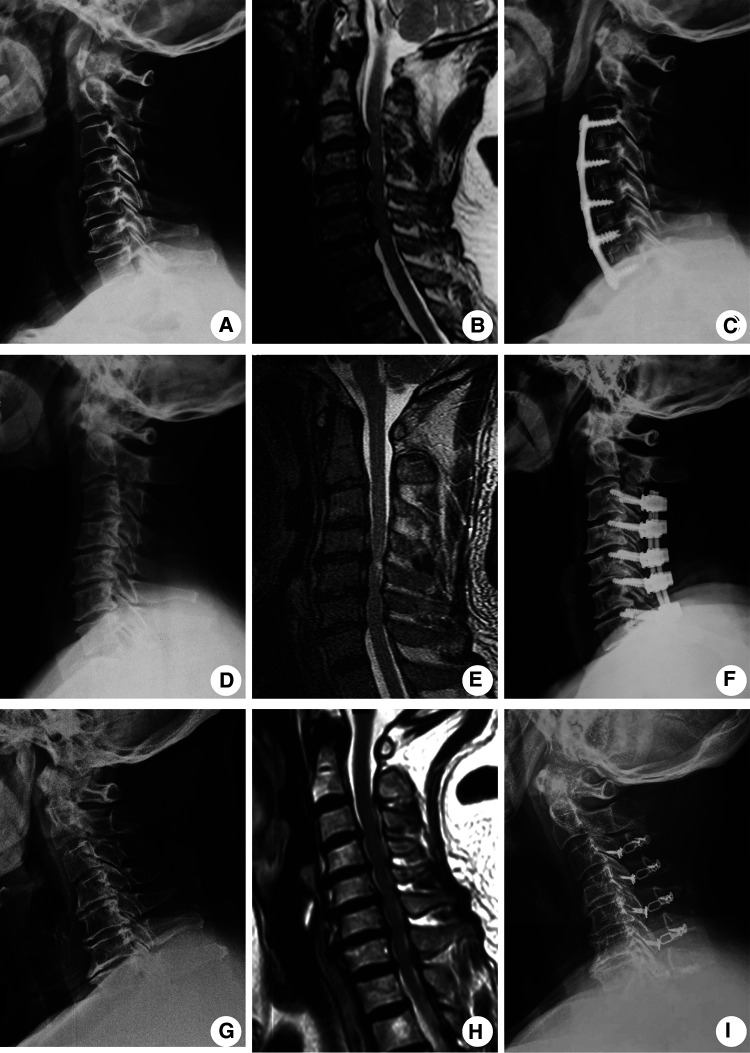
Typical radiological images showing four-level CSM patients treated with either ACDF, LAMP, or the LF approach. Representative preoperative lateral position x-ray radiograph (**A**), preoperative MRI image (**B**), and x-ray radiographs at a 2-year follow-up (**C**) of ACDF-treated patients. Representative preoperative lateral position x-ray radiograph (**D**), preoperative MRI image (**E**), and x-ray radiographs at a 2-year follow-up (**F**) of LF-treated patients. Representative preoperative lateral position x-ray radiograph (**G**), preoperative MRI image (**H**), and x-ray radiographs at a 2-year follow-up (**I**) of LAMP-treated patients.

All operations were performed routinely by two senior surgeons, and the operative procedure was performed as follows (Figure 1).

#### 2.2.1. ACDF (ACDF group)

The ACDF procedure was performed under general anesthesia, with the patients placed in the supine position, the surgical site was exposed through the standard Smith–Robinson approach (10), and ventral compressors of the spinal cord including the intervertebral disc and posterior longitude ligament were removed for direct decompression. The interbody cage (DePuy Spine, USA), combined with the anterior cervical plate (Slim-Loc or SKYLINE, DePuy Spine, USA), was used for anterior fusion (the ACDF group, 38 patients).

#### 2.2.2. Open-door laminoplasty (LAMP group)

After general anesthesia, the patients were placed in the prone position with the head fixed using the Mayfield head holder. Through a posterior midline approach, the lamina and spinous processes were exposed, and the side with relatively severe clinical symptoms and/or radiographic compression was selected as the open side, whose outer and inner cortical margins were both drilled using a high-speed drill. The inner cortical margin of the hinge side was preserved, and the lamina was lifted from the open side toward the hinge side and fixed in an expanded position with 8–12 mm miniplates (LAMP group, 45 patients).

#### 2.2.3. Laminectomy with fusion (LF group)

After general anesthesia, the spinous processes, laminae, facet joints, and transverse processes were exposed through a posterior midline approach that was similar to laminoplasty, and then, lateral mass screws and prebending titanium rods were placed at the planned segment, followed by a resection of the lamina and ligamentum flavum. Autologous bone grafts from the lamina were placed adjacent to bilateral joints to facilitate fusion (LF group, 33 patients).

### 2.3. Clinical evaluation

Baseline data such as demographic information and symptomatology were collected, and operation data on the operation time, blood loss, and hospitalization time were recorded. The Japanese Orthopedic Association scale (JOA), the Neck Disability Index (NDI) scoring system, and the visual analog scale (VAS) scoring system (scores 0–10) evaluating neurological outcomes, neck function, and axial symptoms, respectively, were used for clinical assessment.

### 2.4. Radiological evaluation

**Figure 2:**
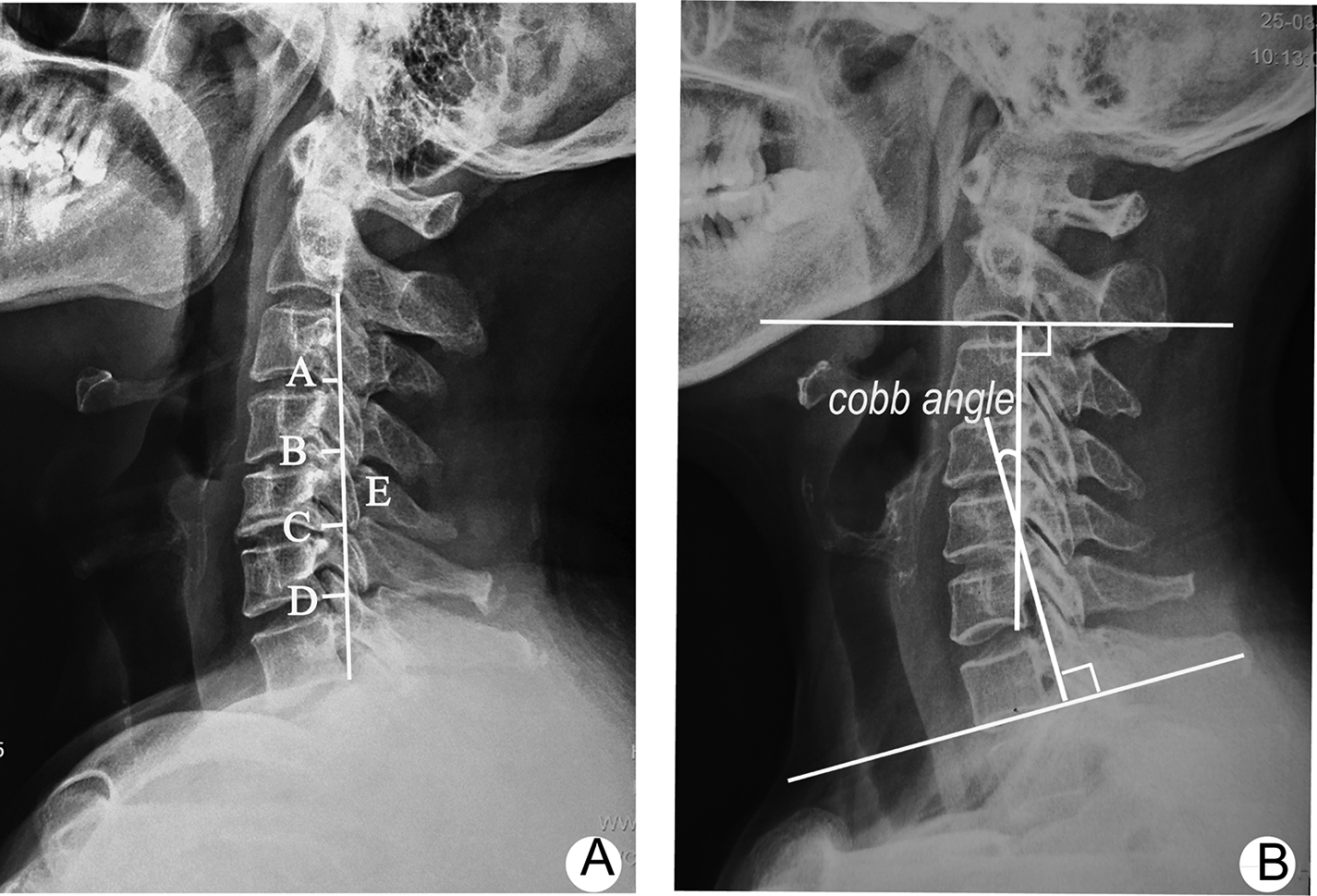
Radiological parameters of the cervical spine. (**A**) CCI measures cervical curvature based on the distance from the posteroinferior edge of the C3–C6 vertebral body to a straight line drawn from the posteroinferior edge of C2–C7 [CI = (A + B + C + D)/E × 100]; (**B**) Cobb angle measures the cervical lordotic angle formed by two lines perpendicular to inferior endplates of the C2 and C7 vertebral bodies, respectively. Cervical range of motion (ROM) was calculated as the difference between the Cobb angles at maximal flexion and extension on anteroposterior (AP) radiographs.

For radiographic assessment, anteroposterior, lateral, and flexion–extension x-ray images of the standing cervical spine were obtained before surgery and during the follow-up period. The cervical curvature index (CCI) and C2–7 Cobb angle evaluating the cervical alignment (Figure 2) and the cervical range of motion (ROM) and active cervical ROM (aROM) evaluating cervical mobility were measured. The aROM was measured using a cervical Range of Motion (ROM) device (Performance Attainment Associates, Roseville, MN, USA). The measurement of the six conventional motions of the cervical spine was performed (flexion, extension, left lateral flexion, right lateral flexion, left rotation and right rotation).

### 2.5. Statistical analysis

Statistical analysis was performed with SPSS version 25.0 (SPSS Inc., Chicago, IL, USA), continuous variables were presented as mean ± standard deviation (SD), and frequencies with percentages were used to summarize categorical variables. The *χ*^2^ test was used for determining categorical variables in demographic data. Fisher's exact test was used for determining categorical variables in postoperative complications. One-way ANOVA, followed by Tukey’s multiple comparison test, was used for determining continuous variables in demographic data as well as clinical and radiographic outcomes. A two-tailed *P* < 0.05 was considered statistically significant.

## 3. Results

### 3.1. Study population

Our cohort consisted of 116 patients (60 males and 56 females) who were followed up for a mean period of 39.4 months (24–72 months) postoperatively. The demographics of the patients are shown in Table 1. No significant differences in age (*P* = 0.098), gender (*P* = 0.625), smoking (*P* = 0.936), diabetes (*P* = 0.974), symptom duration (*P* = 0.472), or follow-up period (*P* = 0.321) were detected among the three groups. The ACDF group had the least bleeding loss, which was in contrast to the LF group, which had the maximum bleeding loss, and the operation time in the LF group was the longest among the three groups, which showed no significant differences between the ACDF and the LAMP groups.

**Table 1:** Demographic and surgical data of patients.

Variables	ACDF group (*n* = 38)	LAMP group (*n* = 45)	LF group (*n* = 33)	*P*-value
Mean age (years)	53.5 ± 10.2	59.4 ± 14.7	58.8 ± 13.9	0.098
Gender (male) (%)	21 (55.26)	25 (55.56)	15 (45.45)	0.625
Current smoker (%)	9 (23.68)	11 (24.44)	9 (27.27)	0.936
Patient with diabetes (%)	7 (18.42)	9 (20.00)	6 (18.18)	0.974
Symptom duration (months)	29.1 ± 11.3	25.7 ± 13.9	26.8 ± 12.4	0.472
Follow-up period (months)	42 ± 20.4	34.8 ± 15.6	38.4 ± 28.8	0.321
Bleeding loss (ml)	102.3 ± 35.8^§¶^	209.3 ± 41.6^†^	343.2 ± 50.5	<0.001
Operation time (min)	95.1 ± 20.6^¶^	102.6 ± 33.4^†^	135.1 ± 37.4	<0.001

The *p*-value was calculated by comparing all groups using one-way ANOVA. ACDF, anterior cervical discectomy and fusion; LAMP, posterior open-door laminoplasty; LF, posterior laminectomy and fusion.

^§^ Statistically significant difference between ACDF and LAMP (*P* < 0.05).

^¶^ Statistically significant difference between ACDF and LF (*P* < 0.05).

^†^ Statistically significant difference between LAMP and LF (*P* < 0.05).

### 3.2. Clinical outcomes

Table 2 summarizes the clinical outcomes of surgery. By assessing the JOA, NDI, and VAS scores before surgery and at the final follow-up, we found no remarkable differences regarding the preoperative clinical symptoms and neurological functions among the three groups (JOA *P* = 0.310, NDI *P* = 0.429, VAS *P* = 0.975). At the final follow-up, all patients achieved significant improvement in JOA, NDI, and VAS scores (*P* < 0.05) (Table 2), However, no differences were detected among the three groups in these scores (ΔJOA *P* = 0.474, ΔNDI *P* = 0.300, and ΔVAS *P* = 0.715).

**Table 2:** Clinical outcomes in each group.

Variables		ACDF group (*n* = 38)	LAMP group (*n* = 45)	LF group (*n* = 33)	*P*-value
JOA	Preoperation	9.26 ± 0.93	9.13 ± 1.26	8.85 ± 1.18	0.310
	Final follow-up	14.22 ± 1.74*	14.85 ± 2.13*	13.97 ± 2.82*	0.198
	ΔJOA	5.12 ± 1.01	5.52 ± 1.57	5.28 ± 1.83	0.474
NDI	Preoperation	36.61 ± 3.52	37.54 ± 4.16	37.77 ± 4.45	0.429
	Final follow-up	14.18 ± 2.25*	13.73 ± 2.57*	14.42 ± 3.74*	0.555
	ΔNDI	−23.14 ± 1.96	−24.08 ± 3.68	−23.79 ± 2.01	0.300
VAS	Preoperation	7.48 ± 3.02	7.54 ± 2.65	7.63 ± 2.70	0.975
	Final follow-up	1.87 ± 1.30*	2.08 ± 1.63*	2.47 ± 1.85*	0.285
	ΔVAS	−5.53 ± 1.97	−5.21 ± 1.35	−5.38 ± 2.04	0.715

The *p*-value was calculated by comparing all groups using one-way ANOVA. ACDF, anterior cervical discectomy and fusion; LAMP, posterior open-door laminoplasty; LF, posterior laminectomy and fusion; JOA, the Japanese orthopedic association scale; NDI, the neck disability index; VAS, the visual analog scale.

* Statistically significant difference between the last follow-up and the preoperative period (*P* < 0.05).

Δ Indicates the change of parameter at the last follow-up compared with the preoperative period.

### 3.3. Radiographic outcomes

The radiographic outcomes were evaluated by analyzing the CCI, C2–C7 Cobb angle, and C2–7 ROM, which are summarized in Table 3. Before surgery, all patients showed no differences in cervical alignment and mobility (CCI *P* = 0.728, C2–C7 Cobb angle *P* = 0.863, C2–7 ROM *P* = 0.448 Table 3). At the final follow-up, only patients in the ACDF group achieved significant improvement in the C2–C7 Cobb angle ranging from (10.0 ± 8.6)° to (17.4 ± 7.9)°, (*P* < 0.05). Simultaneously, the ROM significantly reduced in the ACDF group, which showed identical results in the LAMP and LF groups. Noteworthily, although the ROM decreased in all three groups, LAMP showed a smaller reduction in the ROM; in other words, there was greater preservation of the ROM compared with ACDF and LF.

**Table 3:** Radiographic outcomes in each group.

Variables		ACDF group (*n* = 38)	LAMP group (*n* = 45)	LF group (*n* = 33)	*P*-value
CCI (%)	Preoperation	13.6 ± 7.8	14.8 ± 6.6	14.6 ± 7.1	0.728
	Final follow-up	15.5 ± 7.2	14.3 ± 6.1	14.0 ± 7.3	0.607
	ΔCCI	1.78 ± 6.9	0.98 ± 6.5	1.16 ± 7.0	0.859
C2–C7 Cobb angle (°)	Preoperation	10.0 ± 8.6	9.5 ± 5.9	10.4 ± 7.4	0.863
	Final follow-up	17.4 ± 7.9*	10.1 ± 5.8	10.8 ± 7.0	<0.001
	ΔC2–C7 Cobb angle	7.5 ± 7.7^§¶^	1.1 ± 5.7	0.8 ± 7.1	<0.001
C2–7 ROM (°)	Preoperation	40.4 ± 7.7	38.3 ± 7.6	39.8 ± 8.1	0.448
	Final follow-up	12.8 ± 5.1*	31.6 ± 6.2*	11.5 ± 5.4*	<0.001
	ΔC2–7 ROM	−29.5 ± 6.1^§^	−7.1 ± 6.5^†^	−28.9 ± 7.3	<0.001

The *p*-value was calculated by comparing all groups using one-way ANOVA. ACDF, anterior cervical discectomy and fusion; LAMP, posterior open-door laminoplasty; LF, posterior laminectomy and fusion; CCI, cervical curvature index; ROM, range of motion.

* Statistically significant difference between the last follow-up and the preoperative period (*P* < 0.05).

^§^ Statistically significant difference between ACDF and LAMP (*P* < 0.05).

^¶^ Statistically significant difference between ACDF and LF (*P* < 0.05).

^†^ Statistically significant difference between LAMP and LF (*P* < 0.05).

Δ Indicates the change of parameter at the last follow-up compared with the preoperative period.

### 3.4. Active cervical ROM

The active cervical ROM of all patients in flexion–extension, lateral flexion (left and right), and rotation (left and right) are summarized in Table 4, and the range of flexion–extension, lateral flexion, and total rotation reduced after surgery in the three groups. Comparatively, the LAMP group showed a less reduction of the flexion–extension range (preoperation 102.8 ± 10.9; final follow-up 88.7 ± 11.1) than the ACDF group (preoperation 102.2 ± 10.2; final follow-up 55.1 ± 9.7) and the LF group (preoperation 101.4 ± 11.3; final follow-up 50.6 ± 7.9). Similarly, the preservation of the lateral flexion range in the LAMP group (preoperation 79.4 ± 11.1; final follow-up 66.0 ± 9.8) was greater than that in the ACDF group (preoperation 79.2 ± 11.3; final follow-up 55.7 ± 9.5) and LP group (preoperation 81.7 ± 10.0; final follow-up 54.0 ± 7.4). Furthermore, the total rotation range in the LAMP group (preoperation 123.8 ± 13.2; final follow-up 105.4 ± 10.1) declined to a minimal extent compared with that in the ACDF group (preoperation 127.1 ± 12.6; final follow-up 99.6 ± 10.4) and the LP group (preoperation 121.6 ± 12.8; final follow-up 96.2 ± 9.1) (Table 5). All these results indicate that LAMP was more effective in preserving active cervical ROM than ACDF and LF.

**Table 4:** Active cervical ROM measurement in each group.

Variables		ACDF group (*n* = 38)	LAMP group (*n* = 45)	LF group (*n* = 33)	*P*-value
Flexion–extension	Preoperation	102.2 ± 10.2	102.8 ± 10.9	101.4 ± 11.3	0.852
	Final follow-up	55.1 ± 9.7*	88.7 ± 11.1*	50.6 ± 7.9*	<0.001
	Δ Flexion–extension	−50.8 ± 9.9^§^	−15.1 ± 10.9^†^	−51.6 ± 8.7	<0.001
Lateral flexion	Preoperation	79.2 ± 11.3	79.4 ± 11.1	81.7 ± 10.0	0.545
	Final follow-up	55.7 ± 9.5*	66.0 ± 9.8*	54.0 ± 7.4*	<0.001
	Δ Lateral flexion	−25.3 ± 10.2^§^	−13.6 ± 10.1^†^	−25.9 ± 10.0	<0.001
Total rotation	Preoperation	127.1 ± 12.6	123.8 ± 13.2	121.6 ± 12.8	0.196
	Final follow-up	99.6 ± 10.4*	105.4 ± 10.1*	96.2 ± 9.1*	<0.001
	Δ Total rotation	−27.4 ± 10.5^§^	−17.7 ± 10.8^†^	−25.9 ± 11.2	<0.001

The *p*-value was calculated by comparing all groups using one-way ANOVA. ACDF, anterior cervical discectomy and fusion; LAMP, posterior open-door laminoplasty; LF, posterior laminectomy and fusion; ROM, range of motion.

* Statistically significant difference between the last follow-up and the preoperative period (*P* < 0.05).

^§^ Statistically significant difference between ACDF and LAMP (*P* < 0.05).

^†^ Statistically significant difference between LAMP and LF (*P* < 0.05).

Δ Indicates the change of parameter at the last follow-up compared with the preoperative period.

**Table 5:** Postoperative complications.

Complication	ACDF group (*n* = 38)	LAMP group (*n* = 45)	LF group (*n* = 33)	*P*-value
Dysphagia	6 (15.79%)^§¶^	0 (0.00%)	0 (0.00%)	0.001
Pseudoarthrosis	10 (26.32%)^§¶^	0 (0.00%)^†^	8 (24.24%)	0.001
Hematoma	1 (2.63%)	0 (0.00%)	0 (0.00%)	0.612
Axial pain	3 (7.89%)	4 (8.89%)	8 (24.24%)	0.104
Cerebral fluid leakage	1 (2.63%)	2 (4.45%)	2 (6.06%)	0.857
C5 paralysis	1 (2.63%)	3 (6.67%)	5 (15.15%)	0.163
Infection	0 (0.00%)	0 (0.00%)	1 (3.03%)	0.285
Deterioration in neurologic deficit	1 (2.63%)	1 (2.22%)	0 (0.00%)	>0.999
Revision surgery	7 (18.42%)^§^	1 (2.22%)	4 (12.12%)	0.044
Total^#^	23 (60.53%)^§^	11 (24.44%)^†^	16 (48.49%)	0.003

The *p*-value was calculated by comparing all groups using one-way ANOVA. ACDF, anterior cervical discectomy and fusion; LAMP, posterior open-door laminoplasty; LF, posterior laminectomy and fusion.

^§^ Statistically significant difference between ACDF and LAMP (*P* < 0.05).

^¶^ Statistically significant difference between ACDF and LF (*P* < 0.05).

^†^ Statistically significant difference between LAMP and LF (*P* < 0.05).

^#^ Patients may have had more than one complication, so the total may be less than the sum of categories.

### 3.5. Complications

The postoperative complications showed significant differences among the three groups (*P* = 0.003), with LAMP having a lower total incidence compared with ACDF and LF. As for individual complications, the rates of hematoma, axial pain, cerebrospinal fluid leakage, C5 paralysis, infection, or deterioration in neurologic deficits were comparable among groups. Notably, dysphagia occurred in 15.79% of patients from the ACDF group, which was not observed in the LAMP and LP groups, and the occurrence of pseudoarthrosis showed significant differences in the three groups, with ACDF having the highest rate compared with LAMP, which had no case. In addition, the revision surgery rate in ACDF was remarkably higher than that in LAMP. Taken together, these results indicate that patients who undergo ACDF are more likely to experience dysphagia, pseudoarthrosis, and reoperation than those who are subjected to LAMP, which showed the lowest incidence of postoperative complications.

## 4. Discussion

For surgical management of CSM, it is critical to select the optimal procedure preoperatively, and surgeons should seek adequate nerve decompression, restoring the physiological curvature of the cervical spine, preserving cervical mobility, and reducing postoperative complications as soon as possible.

In the present study, we compared the clinical efficacy of three routinely performed surgical procedures, ACDF, LAMP, and LF, on patients with four-level CSM. By using the JOA, NDI, and VAS score systems, we found that all patients achieved gratifying improvements in clinical symptoms and neurological functions, which showed no significant differences among the three groups. This result indicates that ACDF, LAMP, and LF could offer equal outcomes of nerve decompression. In addition, our operation data showed that ACDF had the least bleeding loss, which was in contrast to LF, which had the maximum bleeding loss. What is more, the operation time in the LF group was the longest among the three groups. This suggests that LF was more invasive and time-consuming but could not exert better nerve decompression than LAMP and ACDF.

The physiological curvature of a healthy cervical spine is characterized as lordosis (11), a large number of patients with CSM, especially four-level CSM, show more or less magnitude of lordosis loss, and the recovery of cervical lordosis affects the long-term clinical outcomes of surgery. In this study, only ACDF rather than LAMP or LF showed improvement on the C2–C7 Cobb angle, whereas this advantage failed to translate into better clinical results at the final follow-up. In addition, CCI, another parameter displaying cervical alignment, showed no difference in all patients between the preoperative period and the final follow-up. These results indicate that, although ACDF had the advantage of restoring the C2–C7 Cobb angle, neither ACDF, LAMP, nor LF affected the cervical curvature in our study. Given that subjects with severe kyphosis were excluded in advance, we attribute this phenomenon to the comparable and relatively mild to moderate change in preoperative cervical alignment.

The cervical spine is a hypermobile structure that allows for flexion, extension, lateral flexion, and rotation (12), and mobility, displayed as the range of motion (ROM), represents the critical physiological function of the cervical spine. Noteworthily, owing to solid fusion or loss of the relevant muscle attachment site, cervical spine surgery often leads to a reduced ROM. Thus, a surgical procedure that carries with it a ROM-preserving advantage seems more likely to bring better long-term outcomes (13). In this study, all patients showed a significant reduction in their cervical ROM at the final follow-up; nevertheless, comparatively, LAMP caused a slighter decrease in cervical mobility. Although no differences were observed in clinical outcomes in this study, we supposed that, with a longer follow-up, the superiority of mobility preservation of LAMP would produce a greater improvement in clinical symptoms and neurological function.

Due to the complex anatomical structures of the cervical spine, whose motion involves multiple vertebral joints simultaneously, it is hard to precisely assess that cervical spine movements rely solely on ROM measurement. Thus, we adopt an active cervical ROM (aROM) using a ROM goniometer, which has been validated as a noninvasive, quick, and reproducible method (14)., The aROM is an important indicator while assessing the recovery of patients with cervical disturbances. Surgical intervention predisposes to a decreased aROM, whereas the degree of reduction varies substantially among different surgical procedures (15, 16). Our previous study reported a decreased aROM after multilevel ACDF (17). In the present study, we measured the aROM in six movement directions for a reliable assessment of cervical mobility (18). We found a significant reduction in all patients at the final follow-up compared with the preoperative period. In addition, the aROM showed similar results to the ROM, which revealed less LAMP reduction than ACDF and LF. Taken together, we can conclude that LAMP is superior to ACDF and LF in terms of cervical mobility preservation.

Postoperative complications are an important indicator for surgical evaluation, which should be considered when selecting surgical procedures. With the development of cervical spine surgery, various relevant complications such as dysphagia, pseudoarthrosis, hematoma, axial pain, cerebrospinal fluid leakage, kyphosis, C5 paralysis, infection, and deterioration in neurologic deficit (19), have been widely reported. The occurrence of complications of cervical spine surgery usually depends on the surgical procedure, segments of operation, and severity of compression on the spinal cord or nerve root (20). For example, dysphagia is a common complication of ACDF, while axial pain is prone to occur after the performance of posterior surgical procedures such as LAMP and LF (21, 22). In the present study, we found a significantly higher incidence of complications in patients who underwent ACDF compared with those who underwent LAMP and LF, especially for dysphagia and pseudoarthrosis. Transient dysphagia was one of the most common postoperative complications following the ACDF procedure (23), a part of which was self-healing, whereas the others may suffer for a long time, severely impacting patients’ living quality. Because the site of operation and fixation with plate and graft were adjacent to the esophagus, patients who underwent the anterior cervical procedure were predisposed to suffer from dysphagia postoperatively, and this tendency was more evident as the operative segments increased (21). Thus, spinal surgeons need to consider this complication when deciding on their surgical procedures. Pseudoarthrosis, by definition, is an undesirable condition in which the intended arthrodesis does not lead to valid fusion, causing local instability (24). Pseudoarthrosis has been widely reported in cervical spine surgery that involves fusion such as ACDF, with roughly approximate occurrences in 30%–50% of cases for three or more levels of ACDF (24). As for LF, most studies indicate higher fusion rates in posterior procedures than in anterior procedures, indicating less occurrence of pseudoarthrosis in posterior procedures (25). Eeric Truumees et al. (26) reported 21.2% of pseudoarthrosis incidence in patients who underwent three or more levels of posterior fusion surgery, which was consistent with our data. As pseudoarthrosis always leads to an instability of cervical biomechanics, a substantial proportion of patients in this study needed revision surgery for further fusion, although some of them were asymptomatic. Noteworthily, LAMP, which does not involve the fusion procedure, showed the least incidence of pseudoarthrosis and revision rates. In this respect, we tend to regard LAMP as the optimal procedure for four-level CSM.

There were some limitations in this present study. Firstly, as a retrospective study with a little sample, our conclusion might be affected by sample selection bias. Secondly, only the efficacy of ACDF, LAMP, and LF on four-level CAM were compared because the number of patients receiving other surgical procedures was too small, which does not necessarily mean that spinal surgeons have to select only one of the three procedures for patients.

## 5. Conclusion

This study systematically compared the efficacy of three routinely performed surgical procedures, ACDF, LAMP, and LF, on patients with four-level CSM, exploring nerve decompression, the restoration of cervical alignment, cervical spine mobility preservation, and postoperative complications. By consulting two-year follow-up data, we observed an equivalent efficacy of ACDF, LAMP, and LF in nerve decompression and symptomatic recovery. Importantly, although ACDF resulted in less bleeding loss and better restoration of the C2–C7 Cobb angle than LAMP or LF, a higher incidence of complications such as dysphagia, pseudoarthrosis, and revision surgery severely limited its application in four-level CSM. In contrast, LAMP showed superiority in terms of preserving cervical mobility and controlling complications compared with ACDF or LF; thus, we prefer recommending LAMP as the optimal surgical procedure for four-level CSM.

## Data Availability

The raw data supporting the conclusions of this article will be made available by the authors, without undue reservation.
